# Initial Defibrillator Pad Position and Outcomes for Shockable Out-of-Hospital Cardiac Arrest

**DOI:** 10.1001/jamanetworkopen.2024.31673

**Published:** 2024-09-09

**Authors:** Joshua R. Lupton, Craig D. Newgard, David Dennis, Jack Nuttall, Ritu Sahni, Jonathan Jui, Matthew R. Neth, Mohamud R. Daya

**Affiliations:** 1 Department of Emergency Medicine, Oregon Health and Science University, Portland; 2 Tualatin Valley Fire and Rescue, Portland, Oregon; 3 Washington County Public Health, Hillsboro, Oregon

## Abstract

**Importance:**

Ventricular fibrillation (VF) or pulseless ventricular tachycardia (pVT) are the most treatable causes of out-of-hospital cardiac arrest (OHCA). Yet, it remains unknown if defibrillator pad position, placement in the anterior-posterior (AP) or anterior-lateral (AL) locations, impacts patient outcomes in VF or pVT OHCA.

**Objective:**

To determine the association between initial defibrillator pad placement position and OHCA outcomes for patients presenting with VF or pVT.

**Design, Setting, and Participants:**

This prospective cohort study included patients with OHCA and VF or pVT treated by a single North American emergency medical services (EMS) agency from July 1, 2019, through June 30, 2023. The study included patients with OHCA treated by a large suburban fire-based EMS agency that covers a population of 550 000. Consecutive patients with an initial EMS-assessed rhythm of VF or pVT receiving EMS defibrillation were included. Pediatric patients (younger than 18 years), interfacility transfers, arrests of obvious traumatic etiology, and patients with preexisting do-not-resuscitate status were excluded.

**Exposure:**

AP or AL pad placement.

**Main Outcomes and Measures:**

Return of spontaneous circulation (ROSC) at any time with secondary outcomes of pulses present at emergency department (ED) arrival, survival to hospital admission, survival to hospital discharge, and functional survival at hospital discharge (cerebral performance category score of 2 or less). Measures included adjusted odds ratios (aOR), multivariable logistic regressions, and Fine-Gray competing risks regression.

**Results:**

A total of 255 patients with OHCA were included (median [IQR] age, 66 [55-74] years; 63 females [24.7%]), with initial pad positioning documented as either AP (158 patients [62.0%]; median [IQR] age, 65 [54-74] years; 37 females [23.4%]) or AL (97 patients [38.0%]; median [IQR] age, 66 [57-74] years; 26 females [26.8%]). Patients with AP placement had higher adjusted odds ratio (aOR) of ROSC at any time (aOR, 2.64 [95% CI, 1.50-4.65]), but not significantly different odds of pulses present at ED arrival (1.34 [95% CI, 0.78-2.30]), survival to hospital admission (1.41 [0.82-2.43]), survival to hospital discharge (1.55 [95% CI, 0.83-2.90]), or functional survival at hospital discharge (1.86 [95% CI, 0.98-3.51]). Competing risk analysis found significantly greater cumulative incidence of ROSC among those at risk with initial AP placement compared with AL (subdistribution hazard ratio, 1.81 [95% CI, 1.23-2.67]; *P* = .003).

**Conclusions and Relevance:**

In this cohort study of patients with OHCA and VF or pVT, AP defibrillator pad placement was associated with higher ROSC compared with AL placement.

## Key Points

**Question:**

What is the optimal initial defibrillator pad placement (anterior-posterior [AP] or anterior-lateral [AL]) for patients presenting with shockable out-of-hospital cardiac arrest (OHCA)?

**Findings:**

In this cohort study of 255 patients with shockable OHCA, patients with defibrillation pads placed AP had 2.64-fold greater odds of return of spontaneous circulation compared with patients with pads placed AL after adjustment for known confounders.

**Meaning:**

These findings suggest that AP placement may be superior to AL placement and clinicians should not assume equivalency of initial defibrillator pad positioning for patients with OHCA presenting with a shockable rhythm.

## Teaser

This cohort study assesses the association between initial defibrillator pad placement position and out-of-hospital cardiac arrest among patients treated by emergency medical services in the United States.

## Introduction

Out-of-hospital cardiac arrest (OHCA) remains a leading cause of mortality.^1^ Ventricular fibrillation (VF) or pulseless ventricular tachycardia (pVT) are the most treatable causes of OHCA, and rapid defibrillation via pads placed in the anterior-posterior (AP) or anterior-lateral (AL) position improves the odds of survival with a good neurologic outcome.^2^ Unfortunately, many patients with shockable initial rhythms (VF or pVT) fail initial defibrillation attempts.^3,4^ Although there has been extensive research on the optimal pad positioning (AP vs AL) for the cardioversion of atrial fibrillation,^5,6,7,8,9,10,11^ there are no prospective studies that have evaluated the optimal initial pad placement for OHCA due to VF or pVT.

A prior retrospective study that compared outcomes by initial pad placement in VF or pVT reported no differences between AP and AL positions, though this study included an AL cohort during a clinical trial (2009 to 2011) compared with a nontrial AP cohort (2006 to 2007), which could have influenced outcomes.^12^ The recently published DOSE-VF study used an initial strategy of AL in all cases, and reported significantly higher rates of return of spontaneous circulation (ROSC) and survival outcomes for patients receiving vector change to AP after 3 failed shocks.^4^ However, it remains unknown if the efficacy was due to the vector change specifically or suggestive that AP may be a better initial vector for shockable OHCA.

Given anatomic differences between the ventricle and atria locations, it is reasonable to question if literature pertaining to pad placement for atrial fibrillation cardioversion also applies to VF or pVT. Our objective was to evaluate the association between initial defibrillator pad-placement (AP vs AL) and OHCA outcomes for patients presenting with VF or pVT.

## Methods

### Study Design

This cohort study followed the Strengthening the Reporting of Observational Studies in Epidemiology (STROBE) reporting guideline. This study was approved by the institutional review board of Oregon Health and Science University. This study was a prospective observational study of patients with OHCA treated by a single emergency medical services (EMS) agency from July, 2019, through June 30, 2023. Details of data abstraction and entry into the registry are included in the eMethods in Supplement 1.

### Study Setting

The study included patients treated by a large suburban fire-based EMS agency in the Portland, Oregon metropolitan region. The catchment area of this EMS agency, covering a population over 550 000, is primarily served by a single-tiered dual-advanced life support (ALS) response system which includes ALS fire first response units and either ALS private ambulance or fire ambulance transport units. Further details on the study setting are included in the eMethods in Supplement 1. Agency protocol recommended placement of pads in the AP position if feasible and vector change to the alternative position, either AL or AP, after 3 consecutive failed shocks. The choice of AP was to allow easier application of vector change, if needed, with the use of mechanical cardiopulmonary resuscitation (CPR) devices in-place. The arrest protocols called for the maximum energy to be used regardless of manufacturer recommendations.

### Patient Population

We included patients with an initial EMS-assessed rhythm of VF or pVT requiring EMS defibrillation, regardless of any pre-EMS automated external defibrillator (AED) defibrillation attempt or placement. We excluded pediatric patients (aged younger than 18 years), interfacility transfers, arrests of obvious traumatic etiology, and patients with preexisting do-not-resuscitate (DNR) status.

### Variables

The primary exposure variable was the initial pad position, which was defined as AP or AL, as documented by the treating EMS clinicians in the PCR. Protocols recommended AP pad placement when possible though final placement decisions were at the discretion of the treating EMS clinicians. Additional variables available included age, sex, arrest location, witness status (unwitnessed, bystander witnessed, EMS witnessed), bystander CPR, bystander AED use, EMS estimated weight, initial defibrillation energy selected, anonymous defibrillator manufacturer, year, and timing elements, including time from the 911 call to EMS arrival on-scene.

### Outcomes

The primary outcome was ROSC at any time. Secondary outcomes included sustained ROSC, defined as pulses present at emergency department (ED) arrival; survival to hospital admission; survival to hospital discharge; and survival to hospital discharge with a good neurologic outcome defined as a Cerebral Performance Category (CPC) score of 2 or less. Process outcomes included the total number of EMS shocks, the proportion of patients achieving ROSC within 20 minutes, the overall time to initial ROSC (regardless if sustained), the time to sustained ROSC (no recurrent arrests through ED arrival), and the need to subsequently change the pad position (vector change). Double sequential defibrillation was not permitted per agency protocol during the study period.

### Statistical Analysis

We used descriptive statistics to evaluate the sample and unadjusted comparisons, including *t* tests and χ^2^ tests. We performed multivariable logistic regressions adjusting for age, sex, arrest location (nonpublic location), witness status (none, bystander, EMS), bystander CPR, bystander AED application, year, and time from 911 call to EMS arrival. We next examined separately any interaction between pad position and EMS-estimated patient weight, initial defibrillator energy level (Joules), and device manufacturer by adding an interaction term to the model. To evaluate differences in the cumulative incidence of ROSC for those still at risk by the primary exposure (AP or AL pad position), we performed a Fine-Gray competing risks regression.^13^ This analysis allowed us to account for a change in pad position (vector change) midway through a resuscitation where thereafter it was impossible to get ROSC as a result of the initial pad position; differences in the time of termination of resuscitation across resuscitations; and differences in time to arrival at the hospital (shorter EMS resuscitation time would reduce the opportunity to achieve ROSC). We coded termination of resuscitation or change in pad position as a competing risk and arrival to the hospital as a censoring event. We performed multiple sensitivity analyses including propensity score matching (eMethods in Supplement 1), using mixed-effects regression to account for clustering by medic unit, and excluding cases with EMS-assessed noncardiac etiologies. We report statistical significance based on an α of .05. All analyses were conducted using Stata version 18.0 (StataCorp). Data were analyzed from February to July 2024.

## Results

**Figure 1.**
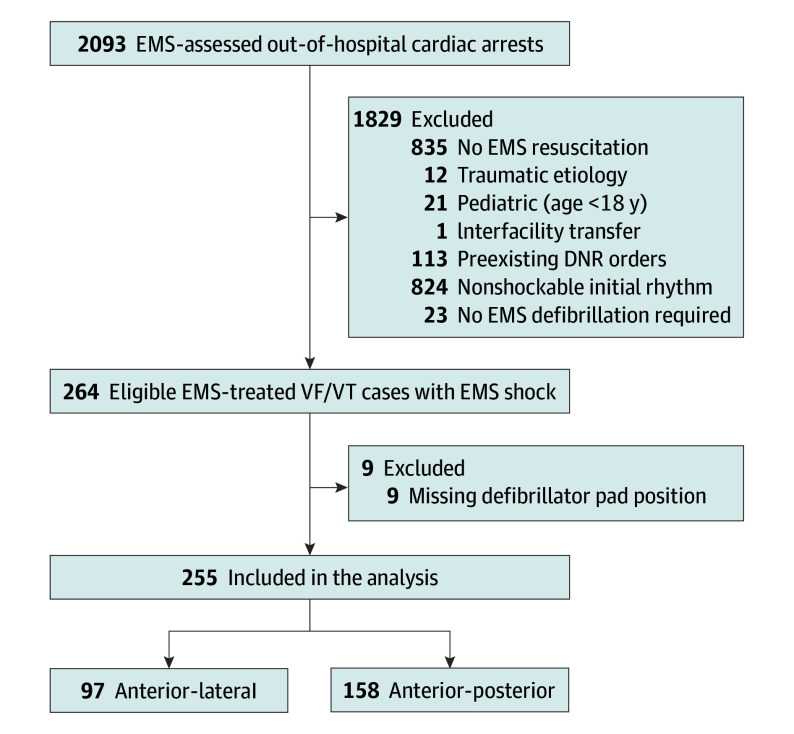
Flow of Inclusion Into the Study Cohort DNR indicates do not resuscitate; EMS, emergency medical services; VF/VT, ventricular fibrillation/ventricular tachycardia.

A total of 255 patients with OHCA were included in the analysis (median [IQR] age, 66 [55-74] years; 63 [24.7%] females), among the 264 eligible for inclusion (Figure 1). Patient characteristics by initial pad placement, AP (158 patients [62.0%]) or AL (97 patients [38.0%]), are summarized in Table 1. There were differences in the distribution of cases by year for AP and AL. There were no statistically significant differences according to pad placement for all baseline characteristics with the exception of EMS-estimated weight (mean [SD] weight: AP, 88.5 [19.5] kg vs AL, 95.2 [33.5] kg; *P* = .05).

**Table 1.** Characteristics of the Study Sample by Initial EMS Pad Positioning

Characteristics	Anterior-posterior (n = 158)	Anterior-lateral (n = 97)
Age, median (IQR), y	65 (54-74)	66 (57-74)
Female	37 (23.4)	26 (26.8)
Male	121 (76.6)	71 (73.2)
Public arrest location	45 (28.5)	24 (24.7)
Witness status		
Unwitnessed	61 (38.6)	38 (39.2)
Bystander witnessed	81 (51.3)	51 (52.6)
EMS witnessed	16 (10.1)	8 (8.2)
Bystander CPR	112 (70.9)	62 (63.9)
Bystander AED applied	28 (17.7)	9 (9.3)
Year		
2019	18 (11.4)	16 (16.5)
2020	37 (23.4)	36 (37.1)
2021	51 (32.3)	24 (24.7)
2022	33 (20.9)	18 (18.6)
2023	19 (12.0)	3 (3.1)
Time from 911 call to EMS arrival, median (IQR), min	4.5 (3.3-5.8)	4.5 (3.2-6.2)
EMS estimated weight, median (IQR), kg	89 (73-100)	91 (75-109)
Initial EMS defibrillation energy, median (IQR), Joules	200 (200-360)	200 (200-360)
Defibrillator manufacturer		
Manufacturer 1	93 (58.9)	67 (69.1)
Manufacturer 2	65 (41.1)	30 (30.9)
EMS contributing arrest etiologies, No. (%)^a^		
Cardiac	142 (89.9)	92 (94.8)
Respiratory	64 (40.5)	32 (33.0)
Suffocation or airway obstruction	3 (1.9)	1 (1.0)
Overdose, toxicologic, or poisoning	5 (3.2)	3 (3.1)
Other	11 (7.0)	7 (7.2)

Abbreviations: AED, automated external defibrillator; CPR, cardiopulmonary resuscitation; EMS, emergency medical services.

^a^ Categories are not mutually exclusive, and EMS could select more than 1 arrest etiology.

Compared with initial AL pad positioning, cases with initial AP placement had a higher unadjusted proportion of ROSC (difference, 23.5% [95% CI, 11.7% to 35.3%]), but no significant differences in pulses present at ED arrival (difference, 8.4% [−4.3% to 21.1%]), survival to hospital admission (difference, 8.9% [−3.8% to 21.6%]), survival to hospital discharge (difference, 8.4% [−3.3% to 20.2%]), and survival to hospital discharge with good neurological function (difference, 11.5% [−0.1% to 23.1%]) as reported in Table 2. After multivariable logistic regression, patients with AP placement had higher adjusted odds (adjusted odds ratio [aOR]) of ROSC at any time (aOR, 2.64 [95% CI, 1.50 to 4.65]), but there was no difference in the odds of pulses at ED arrival (aOR, 1.34 [95% CI, 0.78 to 2.30]), survival to hospital admission (aOR, 1.41 [95% CI, 0.82 to 2.43]), survival to hospital discharge (aOR, 1.55 [95% CI, 0.83 to 2.90]), or survival to hospital discharge with favorable neurological function (aOR, 1.86 [95% CI, 0.98-3.51]). Exploratory process outcomes revealed that AP placement compared with AL had more patients with ROSC within 20 minutes of the 911 call or arrest (if EMS witnessed), a lower proportion with change to a nonshockable rhythm on subsequent rhythm checks, and a lower proportion with receipt of epinephrine at any time (Table 2). Otherwise, there were no significant differences in process outcomes.

**Table 2.** Primary, Secondary, and Process Outcomes by Initial EMS Pad Positioning

Primary and secondary outcomes	Anterior-posterior (n = 158)	Anterior-lateral (n = 97)	*P* value^a^
ROSC			
No. (%)	117 (74.1)	49 (50.5)	<.001
aOR (95% CI)	2.64 (1.50-4.65)	1 [Reference]	.001
Pulses at hospital arrival			
No. (%)	85 (53.8)	44 (45.4)	.19
aOR (95% CI)	1.34 (0.78-2.30)	1 [Reference]	.30
Survival to hospital admission			
No. (%)	89 (56.3)	46 (47.4)	.17
aOR (95% CI)	1.41 (0.82-2.43)	1 [Reference]	.21
Survival to hospital discharge			
No. (%)	54 (34.2)	25 (25.8)	.16
aOR (95% CI)	1.55 (0.83-2.90)	1 [Reference]	.17
Functional survival (CPC ≤2)			
No. (%)	54 (34.2)	22 (22.7)	.05
aOR (95% CI)	1.86 (0.98-3.51)	1 [Reference]	.06
Process outcomes			
Total EMS shocks given, mean (SD)	3.8 (3.5)	4.2 (3.6)	.43
ROSC within 20 min of call or arrest	65 (41.1)	24 (24.7)	.008
Time from call or arrest to:^b^			
First EMS shock, mean (SD), min	9.3 (4.3)	10.0 (4.3)	.20
First ROSC, mean (SD), min	20.4 (10.8)	21.6 (12.6)	.54
Sustained ROSC, mean (SD), min	20.4 (12.5)	25.2 (16.8)	.16
Change to nonshockable arrest rhythm	95 (60.1)	74 (76.3)	.008
Changes to initial EMS pad position	34 (21.5)	28 (28.9)	.18
Use of:			
Any epinephrine	125 (79.1)	88 (90.7)	.02
Any amiodarone	92 (58.2)	59 (60.8)	.68
Repeat amiodarone dosing	48 (30.4)	35 (36.1)	.35
Any advanced airway	130 (83.9)	87 (90.6)	.13
Shock-refractory without organized rhythm^c^	17 (10.8)	18 (18.6)	.08

Abbreviations: aOR, adjusted odds ratio; CPC, cerebral performance category; EMS, emergency medical services; ROSC, return of spontaneous circulation.

^a^ *P* values represent results of *t* tests or χ^2^ testing.

^b^ Time values are from the 911 call (unwitnessed and bystander witnessed) or time of arrest (EMS witnessed).

^c^ Shockable rhythm or asystole throughout without ROSC or organized rhythm (pulseless electrical activity) through entire prehospital care up to hospital care handoff or termination of resuscitation.

**Figure 2.**
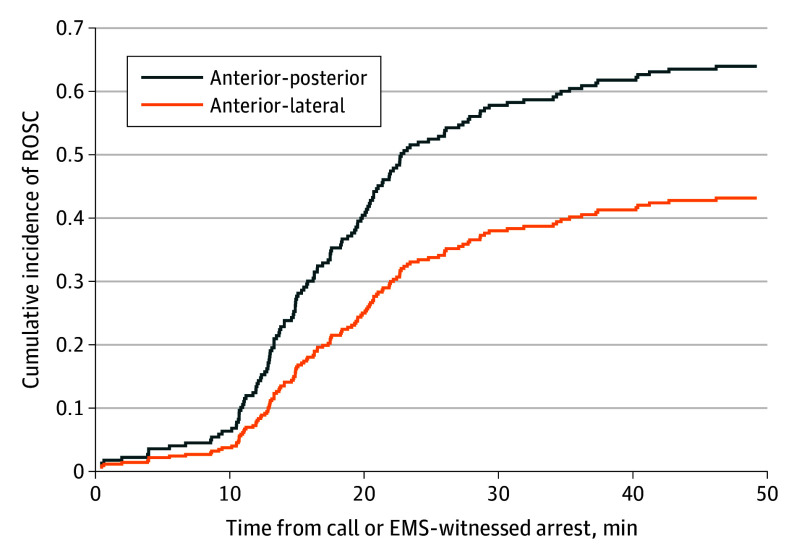
Adjusted Cumulative Incidence of Return of Spontaneous Circulation (ROSC) Throughout the Duration of the Resuscitation Relative to the Time of 911 Call or Arrest EMS indicated emergency medical services.

We next examined differences in the cumulative incidence of ROSC throughout the timeframe of EMS resuscitation relative to the 911 call or time of arrest (if witnessed by EMS) using a competing risks regression and adjusting for the same covariates as in the primary multivariable logistic model. This allowed a specific evaluation of ROSC differences by pad position relative to time at risk by viewing termination of resuscitation or changing pad position as competing risks. This revealed a significantly higher cumulative incidence of ROSC, among those at risk, for patients with AP placement relative to AL (subdistribution hazard ratio, 1.81 [95% CI, 1.23-2.67]; *P* = .003) (Figure 2).

**Figure 3.**
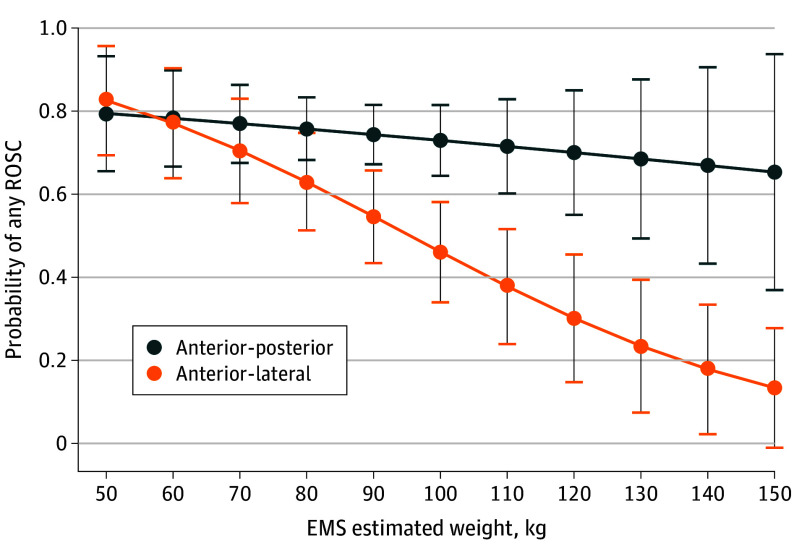
Adjusted Predicted Probabilities of Return of Spontaneous Circulation (ROSC) by Initial Defibrillator Pad Position Over the Range of Emergency Medical Services (EMS)-Estimated Weights Error bars indicate 95% CIs.

We performed several exploratory analyses. We tested for the presence of an interaction between pad placement and patient weight in the adjusted model, which resulted in a significant interaction (interaction OR for AP position × weight of 1.03 [95% CI, 1.00 to 1.06]; *P* = .048), suggesting an increase in the magnitude of the effect size of AP vs AL for increasing weight. This was largely driven by reduced probabilities of ROSC at greater estimated patient weights for the AL pad position (Figure 3). There were no significant interactions between pad positioning and device manufacturer or initial energy selected. To explore any unique differences in AP or AL pad placement among patients who required EMS defibrillation despite already receiving a bystander AED shock, we examined the subset of cases with pre-EMS AED shock attempts. In this small group of 27 patients (11%), EMS AP placement had fewer subsequent EMS shocks (4.1 vs 6.7; difference [95% CI] −2.7 [95% CI, −6.0 to 0.6]) and changes to pad positioning (4 of 20 [20.0%] vs 4 of 7 [57.1%]; difference −37.1% [95% CI, −77.2% to 3.0%]), although these differences were not statistically significant (eTable 1 in Supplement 1).

Finally, we performed several sensitivity analyses to evaluate the robustness of our results. First, we performed propensity score matching, resulting in a matched cohort of 90 patients with AL placement and 90 patients with AP placement, with characteristics reported in eTable 2 in Supplement 1. In this matched cohort, patients with AP placement, compared with AL, had higher ROSC (difference, 15.6% [95% CI, 1.3%-29.8%]; odds ratio, [95% CI], 1.94 [95% CI, 1.05-3.56]), without significant differences in ROSC at ED arrival or survival outcomes (eTable 3 in Supplement 1). To account for any clustering by specific medic unit, we repeated our primary analysis using a mixed-effects model using medic unit as cluster, which did not significantly change the results (eTable 4 in Supplement 1). Excluding the 113 patients with any EMS-assessed noncardiac etiology resulted in 142 patients (58 AL; 84 AP). In this subgroup of cardiac-only etiologies (eTable 4 in Supplement 1), the aOR significantly favored AP, compared with AL, for ROSC (aOR, 2.89 [95% CI, 1.31 to 6.37]), survival to discharge (aOR, 2.86 [95% CI, 1.10 to 7.39]), and favorable neurologic outcome (aOR, 3.26 [95% CI, 1.25 to 8.50]).

## Discussion

We present a prospective observational study evaluating the association between initial defibrillator pad placement (AP vs AL) and patient outcomes in shockable OHCA, with findings suggesting that AP placement was associated with higher ROSC compared with AL. Although patients with AP placement in our cohort required less shocks on average, had earlier initial and sustained ROSC, and required less subsequent changes to pad positioning compared with AL, we were unable to reject the null hypothesis that the differences in these process outcomes were due to chance. Nevertheless, our findings support the critical need for further prospective studies evaluating the optimal initial pad placement for patients in shockable OHCA to maximize the efficacy of early defibrillation and potentially reduce the duration of pulselessness to improve patient survival.

International guidelines on optimal defibrillator pad positioning differ and are largely based on citations from studies of cardioversion for atrial fibrillation.^14,15^ The European Resuscitation Council (ERC) guidelines recommend the AL pad position as the position of choice for initial placement.^15^ The American Heart Association guidelines suggest either AP or AL position are reasonable for defibrillation of VF or pVT, citing studies focused on cardioversion of atrial fibrillation.^14^ Due to the anatomical differences of ventricles compared with the atria, one should not expect studies of atrial fibrillation cardioversion to translate perfectly to VF or pVT. Systematic reviews as well as meta-analysis looking at AP compared with AL for cardioversions of atrial fibrillation have suggested either no difference according to pad positioning^5,6,9^ or concluded that AL is superior to AP.^7,10^ More comparable to VF or pVT defibrillation may be the efficacy of transcutaneous cardiac pacing, with a recent study suggesting that reduced energy was required for capture for with AP placement compared with AL.^16^ Animal models of cardiac arrest and pad placement have also suggested increased efficacy of the AP compared with the AL pad position in a porcine model.^17,18^ Our study adds to this base of evidence and is specific to defibrillation for cardiac arrest patients that present with VF or pVT.

Few studies have evaluated the efficacy of defibrillation by pad position in cardiac arrest patients. A retrospective analysis of AL placement during a clinical trial (2009 to 2011) compared with a nontrial AP historical control placement (2006 to 2007), found no differences in defibrillation efficacy in terminating VF or pVT when assessed 5 seconds after defibrillation.^12^ However, placement (AP or AL) in that study was presumed based on local protocols at the time, rather than specifically documented for each case. Similarly, this study was not able to account for any changes to pad positioning, either initially or during the course of resuscitation. Our study evaluated ROSC rather than VF termination at 5 seconds and accounted for changes to pad positioning through a competing risk analysis to confirm the robustness of our results. The more recent DOSE-VF trial evaluated patients with initial AL placement (control) compared with those switching to the AP position (vector change) or double sequential external defibrillation (DSED) after a failed third defibrillation attempt.^4^ The relative risk of ROSC for the vector change (pads to AP) group relative to continued use of the AL position was 1.39 (95% CI, 0.97-1.99). This is similar to our adjusted odds of ROSC at any time (2.64 [95% CI, 1.50-4.65]) or pulses present at ED arrival (1.34 [95% CI, 0.78-2.30]) for AP compared with AL, particularly given we evaluated outcomes by the initial pad position, rather than comparing a change to AP compared with continued AL in patients meeting shock refractory VF or pVT criteria.

The role of weight in relation to shock success by pad position has also been explored in previous studies evaluating cardioversion of atrial fibrillation, with some suggestion that obese patients have greater success with AL placement.^8,11^ For patients with in-hospital cardiac arrest due to VF or pVT, obesity was associated with a greater number of shocks required prior to achieving ROSC.^19^ Our study found a significant interaction between patient weight and pad position in the association with ROSC with stratified analysis suggesting lower probability of ROSC with initial AL placement in patients with higher body weights compared with initial AP.

Bystander AED placement and use prior to EMS arrival has been associated with improved survival as well as functional outcomes for OHCA.^20,21^ However, patients who fail AED defibrillations and remain in VF or pVT arrest at EMS arrival have a higher prevalence of refractory VF or pVT.^3^ As AEDs are typically placed in the AL position,^22^ we explored if there were any differences in outcomes by EMS pad placement for cases where an AED shock was delivered but unsuccessful, as this may suggest inadequacy of the AL vector. In the small subset of patients in our study who were still in arrest after a bystander AED shock, AP placement by EMS had higher ROSC and functional survival, fewer average shocks delivered by EMS, and fewer instances of subsequent vector change attempts by EMS, though all differences were not statistically significant (eTable 1 in Supplement 1). Taken together with the published data on the potential efficacy of vector change from AL to AP^4^ and the increasing use of AEDs in patients with OHCA prior to EMS arrival, our results highlight the need for future studies on the optimal initial pad position for patients still in VF or pVT after a bystander AED shock.

Our study reported a competing risks time to event analysis for obtaining ROSC by pad positioning after adjusting for known confounders. The advantage of this approach is that it allows us to incorporate the time to event (ROSC) rather than rely on a binary outcome of ROSC, given the association and importance of early ROSC and reduced arrest duration to improved functional outcomes after OHCA. This analysis also allows for some accounting for instances of prolonged field resuscitation (longer time at risk), instances of rapid transfer to the hospital (minimal time at risk), or changes to pad position during the resuscitation. Ultimately, these results aligned well with our primary analysis and supported the association between initial AP pad placement and higher ROSC in VF or pVT OHCA. Future larger studies or trials should explore if initial AP placement leads to an improved functional outcome through a reduction in the time to ROSC compared with initial AL placement.

### Limitations

This study has limitations. Our study was limited by the observational design and there likely remain unmeasured confounders that could bias our results despite adjustment attempts. Additional limitations include remaining selection bias as pad placement was left to the discretion of individual EMS crews, the risk our lack of significance in certain outcomes was due to type II error from being underpowered, and limits to generalizability as our study involves cases treated by a single fire-based EMS agency. Furthermore, use of EMS-estimated weights rather than direct measurement may have biased these comparisons, though previous studies suggest high correlation between EMS-estimated weights and patient weights in cardiac arrest.^23^ Furthermore, use of ROSC as defined by EMS may be complicated by difficulty in pulse palpation in cardiac arrest, which could be exacerbated for patients with greater body weights. Finally, our evaluations of ROSC as a primary outcome are limited because it does not afford an analysis of true termination of VF through a filtered waveform review after each defibrillation attempt. Although this may improve the clinical relevance of our findings, we cannot determine if associated differences in ROSC by pad position are due to differences in the initial termination of VF or pVT if measured immediately after defibrillation.

## Conclusions

In this cohort study of patients with OHCA and VF or pVT, an initial AP defibrillator pad placement was associated with significantly greater adjusted odds of ROSC compared with an initial AL placement. This work emphasizes the critical need for future randomized clinical trials evaluating the optimal initial pad placement for OHCA given the importance of maximizing the efficacy of early defibrillation to achieve timely ROSC and improve neurologically intact survival.

## Notes

Supplement 1.
eMethods.
**eTable 1. **Primary, Secondary, and Process Outcomes by Initial EMS Pad Positioning for Cases With a Pre-EMS AED Shock Delivered **eTable 2. **Characteristics of the Propensity Matched Sample by Initial EMS Pad Positioning **eTable 3. **Primary and Secondary Outcomes by Initial EMS Pad Positioning in the Propensity Matched Cohort **eTable 4. **Outcomes by Initial EMS Pad Positioning—Sensitivity Analyses Adjusting for Clustering by Medic Unit and With Removal of Noncardiac Etiologies

Supplement 2.
Data Sharing Statement
