# Errors in Author Affiliations

**DOI:** 10.1001/jamanetworkopen.2024.48135

**Published:** 2024-11-06

In the Original Investigation titled “Initial Defibrillator Pad Position and Outcomes for Shockable Out-of-Hospital Cardiac Arrest,”^1^ published September 9, 2024, there was an error in the author affiliation for Mr Nuttall and Dr Sahni. Their affiliation should have been Washington County Public Health, Hillsboro, Oregon. This article has been corrected.^1^
