# Supplementary material for: Initial Defibrillator Pad Position and Outcomes for Shockable Out-of-Hospital Cardiac Arrest
Source: JAMA Netw Open. 2024 Sep 9;7(9):e2431673. doi: 10.1001/jamanetworkopen.2024.31673 (PMC11385052; doi:10.1001/jamanetworkopen.2024.31673)
Supplement: Supplement 1. — eMethods. eTable 1. Primary, Secondary, and Process Outcomes by Initial EMS Pad Positioning for Cases With a Pre-EMS AED Shock Delivered eTable 2. Characteristics of the Propensity Matched Sample by Initial EMS Pad Positioning eTable 3. Primary and Secondary Outcomes by Initial EMS Pad Positioning in the Propensity Matched Cohort eTable 4. Outcomes by Initial EMS Pad Positioning—Sensitivity Analyses Adjusting for Clustering by Medic Unit and With Removal of Noncardiac Etiologies [file jamanetwopen-e2431673-s001.pdf]

Supplemental Online Content

Lupton JR, Newgard CD, Dennis D, et al. Initial defibrillator pad position and outcomes for shockable out-of-hospital cardiac arrest. *JAMA Netw Open*. 2024;7(9):e2431673. doi:10.1001/jamanetworkopen.2024.31673

eMethods.
eTable 1. Primary, Secondary, and Process Outcomes by Initial EMS Pad Positioning for Cases With a Pre-EMS AED Shock Delivered
eTable 2. Characteristics of the Propensity Matched Sample by Initial EMS Pad Positioning
eTable 3. Primary and Secondary Outcomes by Initial EMS Pad Positioning in the Propensity Matched Cohort
eTable 4. Outcomes by Initial EMS Pad Positioning—Sensitivity Analyses Adjusting for Clustering by Medic Unit and With Removal of Noncardiac Etiologies

This supplemental material has been provided by the authors to give readers additional information about their work.

© 2024 Lupton JR et al. *JAMA Network Open*.

eMethods.

Cohort Data Abstraction

Trained research assistants preform a review of each case, including verifying imported dispatch and prehospital care record (PCR) data as well as for further abstraction of variables, including from the PCR narrative. Hospital outcomes are obtained through linkage with hospital records. Times are documented during care from a separate tablet with timestamps. At the onset of this cohort, the defibrillation variable in the PCR flowsheet was modified to require emergency medical services (EMS) professionals to input pad position (anterior-posterior [AP] or anterior-latera [AL]) for each defibrillation.

Study Setting

The included cohort is from an EMS agency in a geographic area encompassing 10 incorporated cities across four counties covering an area of 390 square miles and a population over 550,000, making it the second-largest agency in the state. In 2022, the agency responded to over 44,000 calls for medical services including 568 EMS-assessed cardiac arrest cases with 312 EMS-treated cardiac arrest cases (approximately 60 EMS-treated cases per 100,000 inhabitants annually). Standard response for presumed out-of-hospital cardiac arrest (OHCA) incidents includes the dispatch of the two closest advanced life support (ALS) fire units to facilitate high quality cardiopulmonary resuscitation (CPR). Training on proper pad positioning, for both AP and AL, occurred as part of cardiac arrest training every 6 months.

Propensity Score Analysis

© 2024 Lupton JR et al. *JAMA Network Open.*

We conducted propensity score matching as part of a sensitivity analysis to confirm the robustness of our results. We used logistic regression to estimate the propensity score for initial AP pad placement and then matched using nearest neighbor 1:1 matching with a maximum caliper of 0.25 standard deviations from the logit of the propensity score. Covariates used to generate the propensity score included age, sex, arrest location (non-public location), witness status (none, bystander, EMS), bystander CPR, bystander AED application, year, and time from 911 call to EMS arrival. A total of 180 patients were matched, 90 in the AL and 90 in the AP groups. Post-match characteristics, compared to Table 1, are presented in **Supplemental Table 2**, with p-values representing results of t-tests or chi-squared tests of difference as well as standardized mean differences. A standardized mean difference less than 0.1 was considered adequate balance. All variables were adequately balanced, except for AED application, which was higher in the matched AL group than the AP group, and year. There were no significant (all $p>0.05$) differences between characteristics in the matched cohort. Patient outcomes in the matched cohort by pad positioning are reported in **Supplemental Table 3.** Repeating the post-matching analyses using mixed effects regression adjusting for any clustering by year of arrest did not significantly alter the relationships found in the propensity matched cohort.

© 2024 Lupton JR et al. *JAMA Network Open.*

eTable 1. Primary, Secondary, and Process Outcomes by Initial EMS Pad Positioning for Cases With a Pre-EMS AED Shock Delivered

**Primary and Secondary Outcomes**	**Anterior-posterior (AP)** (N=20)	**Anterior-lateral (AL)** (N=7)	**p-value**
Return of Spontaneous Circulation	17 (85.0)	4 (57.1)	0.29
Pulses at Hospital Arrival	12 (60.0)	3 (42.9)	0.66
Survival to Hospital Admission	11 (55.0)	4 (57.1)	>0.99
Survival to Hospital Discharge	6 (30.0)	0 (0.0)	0.16
Functional Survival (CPC ≤2)	6 (30.0)	0 (0.0)	0.16
**Process Outcomes**	**Anterior-posterior (AP)** (N=20)	**Anterior-lateral (AL)** (N=7)	**p-value**
Total EMS shocks given, mean (sd)	4.1 (3.3)	6.7 (4.5)	0.11
ROSC within 20 minutes of call or arrest	9 (45.0)	1 (14.3)	0.20
Time from call or arrest to:			
1st EMS shock (minutes), mean (sd)	9.6 (1.8)	11.9 (2.8)	0.02
1st ROSC (minutes), mean (sd)	20.6 (7.8)	23.7 (5.5)	0.45
Sustained ROSC (minutes), mean (sd)	26.1 (14.3)	34.9 (21.4)	0.41
Changes to initial EMS pad position	4 (20.0)	4 (57.1)	0.15

Time values are from the 911 call (unwitnessed and bystander witnessed) or time of arrest (EMS witnessed). P-values represent results of t-tests or Fisher exact testing. Abbreviations: CPC = cerebral performance category; aOR = adjusted odds ratio; 95% CI = 95% confidence intervals; EMS = emergency medical services; ROSC = return of spontaneous circulation.

© 2024 Lupton JR et al. *JAMA Network Open.*

eTable 2. Characteristics of the Propensity Matched Sample by Initial EMS Pad Positioning

	**Anterior-posterior (AP)** (n=90)	**Anterior-lateral (AL)** (n=90)	**p-value**	**Standardized Mean Difference**
Age – years (median [IQR])	66 (53-76)	66 (56-72)	0.66	-0.07
Female	25 (27.8)	23 (25.6)	0.87	0.05
Male	65 (72.2)	67 (74.4)		
Public Arrest Location	24 (26.7)	22 (24.4)	0.86	0.05
Witness Status				
Unwitnessed	34 (37.8)	37 (41.1)	0.82	0.09
Bystander Witnessed	46 (51.1)	45 (50.0)		
EMS Witnessed	10 (11.1)	8 (8.9)		
Bystander CPR	57 (63.3)	60 (66.7)	0.76	0.07
Bystander AED Applied	6 (6.7)	9 (10.0)	0.59	0.12
Year				
2019	14 (15.6)	16 (17.8)	0.78	0.21
2020	35 (38.9)	29 (32.2)		
2021	21 (23.3)	24 (26.7)		
2022	19 (21.1)	18 (20.0)		
2023	1 (1.1)	3 (3.3)		
Time from 911 Call to EMS Arrival – minutes (median [IQR])	4.4 (3.1-5.7)	4.5 (3.3-6.3)	0.96	0.01

Abbreviations: EMS = emergency medical services; CPR = cardiopulmonary resuscitation; AED = automated external defibrillator; IQR = interquartile range

© 2024 Lupton JR et al. *JAMA Network Open.*

eTable 3. Primary and Secondary Outcomes by Initial EMS Pad Positioning in the Propensity Matched Cohort

**Primary and Secondary Outcomes**		**Anterior-posterior (AP)** (N=90)	**Anterior-lateral (AL)** (N=90)	**p-value**
Return of Spontaneous Circulation	n (%)	62 (68.9)	48 (53.3)	0.03
	OR (95% CI)	1.94 (1.05-3.56)	reference	
Pulses at Hospital Arrival	n (%)	46 (51.1)	43 (47.8)	0.66
	OR (95% CI)	1.14 (0.64-2.05)	reference	
Survival to Hospital Admission	n (%)	48 (53.3)	43 (47.8)	0.46
	OR (95% CI)	1.25 (0.70-2.24)	reference	
Survival to Hospital Discharge	n (%)	30 (33.3)	25 (27.8)	0.42
	OR (95% CI)	1.30 (0.69-2.46)	reference	
Functional Survival (CPC ≤2)	n (%)	30 (33.3)	22 (24.4)	0.19
	OR (95% CI)	1.55 (0.81-2.96)	reference	

Abbreviations: CPC = cerebral performance category; aOR = adjusted odds ratio; 95% CI = 95% confidence intervals; EMS = emergency medical services; ROSC = return of spontaneous circulation.

© 2024 Lupton JR et al. *JAMA Network Open.*

eTable 4. Outcomes by Initial EMS Pad Positioning—Sensitivity Analyses Adjusting for Clustering by Medic Unit and With Removal of Noncardiac Etiologies

**Outcomes**	**Adjusted odds ratio (95% CI) for Anterior-posterior vs. Anterior-lateral (reference)**	
	**Medic unit as cluster**	**Only cardiac etiology (n=142)**
Return of Spontaneous Circulation	2.72 (1.50-4.92)	2.89 (1.31-6.37)
Pulses at Hospital Arrival	1.34 (0.78-2.30)	1.30 (0.60-2.80)
Survival to Hospital Admission	1.40 (0.80-2.44)	1.99 (0.92-4.30)
Survival to Hospital Discharge	1.49 (0.78-2.86)	2.86 (1.10-7.39)
Functional Survival (CPC ≤2)	1.75 (0.89-3.43)	3.26 (1.25-8.50)

Abbreviations: CPC = cerebral performance category; aOR = adjusted odds ratio; 95% CI = 95% confidence intervals; EMS = emergency medical services; ROSC = return of spontaneous circulation.

© 2024 Lupton JR et al. *JAMA Network Open.*
