# Supplementary material for: Initial Defibrillator Pad Position and Outcomes for Shockable Out-of-Hospital Cardiac Arrest
Source: JAMA Netw Open. 2024 Sep 9;7(9):e2431673. doi: 10.1001/jamanetworkopen.2024.31673 (PMC11385052; doi:10.1001/jamanetworkopen.2024.31673)
Supplement: Supplement 2. — Data Sharing Statement [file jamanetwopen-e2431673-s002.pdf]

Data Sharing Statement

Lupton. Initial Defibrillator Pad Position and Outcomes for Shockable Out-of-Hospital Cardiac Arrest. *JAMA Netw Open*. Published September 09, 2024.
doi:10.1001/jamanetworkopen.2024.31673

Data
Data available: No
